# Interformat Reliability of Digital Psychiatric Self-Report Questionnaires: A Systematic Review

**DOI:** 10.2196/jmir.3395

**Published:** 2014-12-03

**Authors:** Sven Alfonsson, Pernilla Maathz, Timo Hursti

**Affiliations:** ^1^U-CAREDepartment of Public Health and Caring SciencesUppsala UniversityUppsalaSweden; ^2^U-CAREDepartment of PsychologyUppsala UniversityUppsalaSweden; ^3^Department of PsychologyUppsala UniversityUppsalaSweden

**Keywords:** psychometric, reliability, questionnaire, psychotherapy, computer, Internet

## Abstract

**Background:**

Research on Internet-based interventions typically use digital versions of pen and paper self-report symptom scales. However, adaptation into the digital format could affect the psychometric properties of established self-report scales. Several studies have investigated differences between digital and pen and paper versions of instruments, but no systematic review of the results has yet been done.

**Objective:**

This review aims to assess the interformat reliability of self-report symptom scales used in digital or online psychotherapy research.

**Methods:**

Three databases (MEDLINE, Embase, and PsycINFO) were systematically reviewed for studies investigating the reliability between digital and pen and paper versions of psychiatric symptom scales.

**Results:**

From a total of 1504 publications, 33 were included in the review, and interformat reliability of 40 different symptom scales was assessed. Significant differences in mean total scores between formats were found in 10 of 62 analyses. These differences were found in just a few studies, which indicates that the results were due to study effects and sample effects rather than unreliable instruments. The interformat reliability ranged from *r*=.35 to *r*=.99; however, the majority of instruments showed a strong correlation between format scores. The quality of the included studies varied, and several studies had insufficient power to detect small differences between formats.

**Conclusions:**

When digital versions of self-report symptom scales are compared to pen and paper versions, most scales show high interformat reliability. This supports the reliability of results obtained in psychotherapy research on the Internet and the comparability of the results to traditional psychotherapy research. There are, however, some instruments that consistently show low interformat reliability, suggesting that these conclusions cannot be generalized to all questionnaires. Most studies had at least some methodological issues with insufficient statistical power being the most common issue. Future studies should preferably provide information about the transformation of the instrument into digital format and the procedure for data collection in more detail.

## Introduction

The use of computerized psychological assessment has increased, and today many patients are also helped through Internet-based psychological interventions. The efficacy of Internet-based interventions has been evaluated repeatedly, and the effects seem to be comparable to live interventions [1-3]. When collecting psychiatric data through computers or evaluating Internet interventions, researchers typically rely on digital versions of existing pen and paper (PnP) self-report instruments. However, it cannot be assumed that instruments retain their psychometric properties when the format of delivery is changed [4]. The level of equality between different delivery formats is here referred to as *interformat reliability*. A high interformat reliability indicates that the psychometric properties of the instrument are independent of the delivery format.

Interformat reliability could be affected in two main ways: by characteristics of the delivery format itself or by how respondents perceive the delivery format. Digital instruments can be presented on different platforms, for example, on a standalone computer, an online Web page, or a mobile phone. Each platform has its own interface, and scores could be affected as a result. The presentation of an instrument can also be different in other ways, for example, by presenting one item at a time as opposed to several items on the same page. Effects of interface and presentation of digital instruments have not been investigated empirically to any large extent, making the strength of such possible effects unclear [5,6]. Since the effects are uncertain, design choices regarding the adaptation of instruments to digital format are important. For example, it can be argued that differences in layout adaptation may affect the validity of the results [4].

Further, people might respond differently to a digital instrument depending on how they perceive the level of security and anonymity [7]. Some people may also feel uncomfortable in using digital devices, which may affect the results [8]. People may express themselves differently in a digital context, for example, when communicating via the Internet, as compared to face-to-face interactions [8,9]. For example, some very sensitive data may benefit from digital assessment [10,11]. If respondents’ ratings on items and the resulting score are affected by presentation format, this could affect the conclusions drawn from Internet-based psychotherapy research.

Thus, investigations of the psychometric equivalence of computer- and PnP-administered instruments are warranted. Quite a few examinations of the interformat reliability of self-report questionnaires have been done, especially among somatic patients, but no systematic review focusing on psychiatric instruments has yet been conducted [12]. Such a review would be valuable in deciding whether transformation of questionnaires to online use are feasible and whether scores from pen and paper and digital versions can be compared.

The objective of this study was to review the interformat reliability of self-report symptom scales used in psychotherapy research. This review also aimed to assess the methodological quality of studies investigating interformat reliability.

## Methods

### Search Strategy

The review process was guided by the Cochrane Handbook of Interventions and the Preferred Reporting Items for Systematic Reviews and Meta-Analyses (PRISMA) guidelines. Unfortunately, there are no specific guidelines for reviewing clinical measurement tools, such as self-report instruments, and the recommendations for judging risk of bias cannot be applied directly. As a consequence, a protocol for quality assessment was created for this review, based on the guidelines in the Cochrane handbook. A systematic search of the literature was performed in the databases MEDLINE, Embase, and PsycINFO. The search strategy included four concepts: the digital format, self-report questionnaires, psychometric properties, and psychology. Several search terms were used for each concept; for example, the search line in MEDLINE was “computer OR Internet OR online AND questionnaire OR instrument OR scale AND psychometric OR reliability OR validity AND psychology OR psychotherapy OR psychological”. The reference lists of included publications were examined in order to identify additional relevant studies. The risk of bias may be high in such perusal of reference lists and is often discouraged. In the present case, the risk of bias was judged somewhat lower and counterbalanced against the benefit of finding older studies and papers published outside the usual channels. No attempt was made to locate unpublished material. The literature search was conducted between January and May 2013, and to identify any studies published on a later date, an additional search was done in January 2014.

### Study Selection

All published peer-reviewed English language research studies comparing the psychometric properties of computerized and PnP versions of self-report instruments were considered for inclusion in the review. Study subjects had to be adults and data on interformat reliability reported as part of the results, either as a correlation, an analysis of differences between format mean scores, or as comparison between theoretical models.

Studies investigating instruments designed to measure symptoms of any of the following Diagnostic and Statistical Manual of Mental Disorders, Fourth Edition (DSM-IV) Axis I group of diagnoses were included: mood disorders, anxiety disorders, eating disorders, substance use disorders, and sleep disorders. Instruments assessing personality traits or non-clinical behaviors (eg, exercise) were excluded. Only instruments of the questionnaire type with set answers and ratings were included; for example, not behavioral assessments, Visual Analogue Scale (VAS), diaries, or open questions. Also, only studies of instruments that previously had been psychometrically assessed in pen and paper format were included.

After the initial search, the title and abstract of identified publications were examined by the first and second authors independently. Irrelevant publications were subsequently excluded. All publications judged relevant were retrieved in full text. The first and second author independently reviewed all full text publications based on the inclusion and exclusion criteria. In case of disagreements, consensus was sought through discussion. If agreement could not be reached, the third author’s judgment was final.

### Data Extraction Procedure

A systematic approach to data extraction was used to produce a descriptive summary of the methods used and the psychometric findings in each included study. The study characteristics (year of publication, sample size, administration format [computer, online, or palm/cell phone], design) and participant characteristics (population, age, gender, computer experience) were extracted. Psychometric data regarding each instrument’s interformat reliability, as well as regarding test-retest reliability and internal consistency of the digital format, were extracted from each study. The first and second author independently extracted data from all included studies.

### Quality Assessment

There are no established guidelines for assessing the quality of psychometric studies, and so a strategy was created for this study. The quality of each study was assessed and rated on six aspects: (1) type of analysis used to compare instruments, (2) use of a randomization procedure, (3) reporting of statistics and results, (4) sample size, (5) sample type, and (6) description of digital instrument adaptation and design. Each aspect was rated on a 3-point scale (0-2), providing a quality score with a possible range from 0-12. See Multimedia Appendix 1 for detailed descriptions of quality assessment. The first and second authors assessed each study independently and agreed on 90.3% of the quality assessment elements before discussion. Any remaining disagreement was discussed with the last author whose judgment was decisive. Studies obtaining a total score above two thirds of the maximum score (ie, >8) were considered high quality studies.

### Study Designs

Interformat reliability can be investigated using either a one sample design or two samples design. Both designs can be further enhanced by adding randomization and crossover design elements. In one sample designs, a single sample is drawn from a population. Data for different formats of an instrument can then be collected by randomizing participants to either complete a PnP or a digital version of the instrument, or by letting each participant complete the instrument in both formats, that is, a crossover design. In the simpler forms of one sample design, participants either first complete one format and then the other without randomizing the order of formats, or are allocated to a group that completes only one of the two formats. These designs have major weaknesses since order effects cannot be separated, and there may be group differences. Hence, there is a clear risk of bias and randomized crossover design is to be preferred. Crossover design has the additional advantage of providing greater statistical power and thus requiring a smaller sample size. In a two samples design, two samples are drawn from one or two different populations. Participants in one of the samples complete the instrument in the PnP version, while participants in the other sample complete the instrument in the digital version. Since mean scores and variance cannot be assumed to be equal in two samples or populations, any conclusions about interformat reliability drawn from a study using a two sample design will be without scientific value.

An additional way to assess interformat reliability is to investigate whether the statistical model of an instrument is equal in both formats. When an instrument is designed, item- or factor analysis is often conducted. If the structure of the instrument can be replicated in the digital version, this will provide some evidence of interformat reliability. However, the structure can be equal in both formats while the actual scores on the instrument diverge, and therefore this should be used only as an addition to other analyses of equivalence.

### Assessing Interformat Reliability

To be able to compare results from instruments in digital format with results from PnP format, one must investigate either correlations between scores or differences between mean scores. Reliability is typically measured with correlation analysis where a result closer to 1 is a stronger relationship [13]. While Pearson correlation is sometimes used when analyzing reliability, for interformat reliability intraclass correlation is more adequate. It is important to note that a high correlation between two scores does not imply that the scores are at the same level. For example, one score could be systematically lower than the other and the correlation would still be high. Hence, equality between different formats is better assessed with a statistical test of differences between group mean scores, for example, by *t* tests or analysis of variance (ANOVA). A study comparing instruments in two different formats should thus report both correlations and analysis of differences between mean scores, with the latter being essential.

Other forms of reliability commonly investigated in instruments are test-retest and internal reliability [13]. As with interformat reliability, it cannot be assumed that test-retest or internal reliability of the instrument is sustained when the format of delivery is changed.

### Sample Size and Effect Size

Sample size calculations should be based on analysis of mean differences, something that typically requires larger sample sizes than correlation analysis. When calculating power and sample size for a study, one should decide how large differences between formats one is ready to accept. While many studies of Internet-based psychological interventions show medium to large effect sizes, it could be argued that when it comes to differences between instrument formats, even small effect sizes are important to detect. For correlation analysis, sample size calculations should be based on achieving adequate confidence intervals rather than significance testing [14].

### Statistical Analyses

When statistical analyses of mean differences were not reported in the original publication, the authors performed the corresponding *t* test if required data were available. The authors also calculated effects sizes for differences where these were not reported in the original publications. Differences between groups of studies (ie, high and low quality studies) were analyzed with *t* tests or Mann-Whitney tests. Interformat reliability on an aggregate level was investigated by comparing mean scores and using calculations of binominal probability. Cohen’s *d* was used as a measure of effect size where .2 is considered a small effect, .5 a medium effect, and .8 a large effect size. A *P* value of <.05 was used as a threshold of statistical significance.

## Results

### Identified and Included Publications

The initial search yielded a total of 1504 hits in the databases. After review of titles and abstracts, 61 publications were selected for full text review. Of these 61 publications, 29 met inclusion criteria. An additional 8 publications were included after the examination of reference lists. Following the additional search, one more publication was included. In total, 38 publications were thus included for data extraction. A complete list of included studies [15-52] can be found in Table 1. A PRISMA flow diagram of the search and inclusion process can be found in Multimedia Appendix 2.

### Data Extraction

Before discussion, reviewers agreed on 92.6% of extracted data elements regarding the study characteristics, 90.3% of the quality assessment elements, and 99.3% of the psychometric data. This is considered a high level of agreement. When studies did not report analysis of mean differences between formats, *t* tests were calculated by the reviewers to complete the results. When crossover studies did not report total format mean scores, this was calculated by the reviewers, based on the reported group mean scores. When two sample designs are used, equality between mean scores of the instruments cannot be assumed. Therefore, the five studies using this design were excluded for further analyses of interformat reliability but are included in Table 1 for the benefit of readers.

### Study Characteristics

The included 33 publications were published between 1985 and 2013. All publications described unique studies, and most studies investigated more than one instrument. Sample sizes ranged from 29 to 1171 (mean 224, SD 277.5). A third of studies (11/33, 33%) assessed stand-alone computer administration, more than half (19/33, 58%) assessed online administration, and very few assessed palm device administration (2/33, 6%) and both online and smartphone administration (1/33, 3%). Nearly a quarter of the studies (8/33, 24%) included some assessment of computer experience. The included studies investigated 40 different self-report instruments, covering the following diagnoses or problem areas: Panic disorder, Depression, Anxiety, Eating disorders, Alcohol and tobacco dependence or misuse, Obsessive Compulsive Disorder, Posttraumatic stress, Postpartum Depression, Social Anxiety Disorder, Insomnia, and perceived physical and mental health.

### Participant Characteristics

We found that 42% (14/33) of studies used a sample from patients or other appropriate population, 45% (15/33) used a student sample, 9% (3/33) used some kind of community sample, and 3% (1/33) did not define the sample. The mean age of the participants ranged between 18.8 and 68.3 years. The gender proportions of the samples ranged from 23.9% to 79.9% women in the studies that included both sexes. Two studies investigated screening instruments of postpartum depression and used all women samples. See Multimedia Appendix 3 for a complete list of study and participant characteristics.

### Design and Quality of Included Studies

Of the 33 studies, 17 (52%) employed a crossover design. Most studies (29/33, 88%) reported adequate statistics, while more than half (17/33, 52%) did not describe the adaptation of the instruments to a digital format. The mean quality score was 8.6. Using a cut score of >8 (two thirds of total quality score), 20 of the 33 studies (61%) were assessed as high quality studies. See Multimedia Appendix 1 for complete quality assessment scores.

**Table 1:** Included publications, investigated instruments, formats compared, and study designs.

Publication	Instruments	Formats	Design
Austin et al (2006) [15]	Body Sensations Questionnaire, Agoraphobic Cognitions Questionnaire, Mobile Inventory	PnP, online	One sample crossover (2x2)
Brock et al (2012) [16]	Center for Epidemiological Studies-Depression, Beck Anxiety Inventory	PnP, online	One sample crossover (4x2)
Bush et al (2013) [17]	PTSD Check List–Civilian Version, Patient Health Questionnaire-9	PnP, online, smartphone	One sample randomized
Butler et al (1988) [18]	Setting Conditions for Anorexia Nervosa Scale	PnP, computer	One sample crossover (2x2)
Carlbring et al (2007) [19]	Body Sensations Questionnaire, Agoraphobic Cognitions Questionnaire, Mobile Inventory, Beck Anxiety Inventory, Beck Depression Inventory-II, Montgomery-Asberg Depression Rating Scale-Self-report	PnP, online	One sample crossover (2x2)
Chan-Pensley (1999) [20]	Alcohol Use Disorder Identification Test	PnP, computer	One sample crossover (2x2)
Coles et al (2007) [21]	Obsessive-Compulsive Inventory, Obsessive Beliefs Questionnaire-44	PnP, online	One sample crossover (2x2)
Cook et al (2007) [22]	Quick Inventory of Depressive Symptomatology Self Rated	PnP, palm	One sample crossover (2x2)
Fortson et al (2006) [23]	Center for Epidemiologic Studies Depression scale, Trauma Symptom Screen	PnP, online	One sample crossover (4x2)
George et al (1992) [24]	Beck Depression Inventory, State-Trait Anxiety Inventory-State, State-Trait Anxiety Inventory-Trait	PnP, computer	One sample randomized
Glaze & Cox (1991) [25]	Edinburgh Postnatal Depression Scale	PnP, computer	One sample crossover (2x2)
Herrero & Meneses (2006) [26]	Center for Epidemiologic Studies Depression scale -7	PnP, online	One sample randomized
Hirai et al (2011) [27]	Social Interaction Anxiety Scale, Social Phobia Scale	PnP, online	One sample randomized
Holländare et al (2008) [28]	Beck Depression Inventory-II, Montgomery-Asberg Depression Rating Scale-Self-report	PnP, online	One sample crossover (2x2)
Holländare et al (2010) [29]	Beck Depression Inventory-II, Montgomery-Asberg Depression Rating Scale-Self-report	PnP, online	One sample crossover (2x2)
Kurt et al (2004) [30]	Center for Epidemiologic Studies Depression scale -R-20, Geriatric Depression Scale-15	PnP, computer	One sample crossover (2x2)
Lankford et al (1994) [31]	Beck Depression Inventory, State-Trait Anxiety Inventory	PnP, computer	One sample
Lukin et al (1985) [32]	Beck Depression Inventory, State-Trait Anxiety Inventory	PnP, computer	One sample crossover (2x2)
Miller et al (2002) [33]	Alcohol Use Disorder Identification Test, Alcohol Dependence Scale, Rutgers Alcohol Problem Index	PnP, online	One sample randomized
Murelle et al (1992) [34]	Michigan Alcohol Screening Test, CAGE Substance Abuse Screening Tool, Fagerstrom Tolerance Questionnaire, Center for Epidemiologic Studies Depression scale, Eating Attitudes Test, Drug Abuse Screen Test, State-Trait Anxiety Inventory	PnP, computer	One sample non random
Ogles et al (1998) [35]	Center for Epidemiologic Studies Depression scale	PnP, computer	One sample non random
Read et al (2008) [36]	PTSD Check List–Civilian Version, Traumatic Life Events Questionnaire	PnP, online	One sample non random
Schulenberg & Yutrzenka (2001) [37]	Beck Depression Inventory-II	PnP, computer	One sample crossover (4x2)
Schmitz et al (2000) [38]	Symptom Checklist 90 Revised	PnP, computer	One sample randomized
Swartz et al (2007) [39]	Center for Epidemiologic Studies Depression scale	PnP, PDA	One sample crossover (2x2)
Thorén et al (2012) [40]	Hospital Anxiety and Depression Scale	PnP, online	One sample crossover (2x2)
Thorndike et al (2011) [41]	Insomnia Severity Index	PnP, online	One sample crossover (2x2)
Vallejo et al (2007) [42]	General Health Questionnaire-28, Symptom Checklist 90 Revised	PnP, online	One sample non-random
Vallejo et al (2008) [43]	General Health Questionnaire-28, Symptom Checklist 90 Revised	PnP, online	One sample crossover (2x2)
Whitehead (2011) [44]	Hospital Anxiety and Depression Scale	PnP, online	One sample randomized
Wijndaele et al (2007) [45]	General Health Questionnaire-12, Symptom Checklist 90 Revised	PnP, online	One sample non random
Yu & Yu (2007) [46]	Center for Epidemiologic Studies Depression Scale	PnP, online	One sample randomized
Zimmerman & Martinez (2012) [47]	Clinically Useful Depression Outcome Scale	PnP, online	One sample non random
Andersson et al (2003) [48]	Hospital Anxiety and Depression Scale	PnP, online	Two samples
Hedman et al (2010) [49]	Liebowitz Social Anxiety Scale Test Self-Report, Social Phobia Scale, Social Interaction Anxiety Scale, Montgomery-Asberg Depression Rating Scale-Self-report, Beck Anxiety Inventory	PnP, online	Two samples
Le et al (2009) [50]	Panic Disorder Severity Scale, Edinburgh Postnatal Depression Scale	PnP, online	Two samples
Schmitz et al (1999) [51]	Symptom Checklist 90 Revised	PnP, computer	Two samples
Shea et al (2009) [52]	Depression Anxiety Stress Scale-21	PnP, online	Two samples

### Interformat Reliability

Including the analyses conducted by the authors of this review, differences between mean scores were analyzed in 88% (29/33) of the studies; 52% (17/33) also had adequate power to detect differences of at least moderate effect size. Subscales were excluded in the analysis of interformat reliability since data from some instruments with several subscales, notably the Symptom Checklist 90 Revised (SCL-90R) and the General Health Questionnaire (GHQ-28), would have a disproportional influence on the results. Focusing on the total scores of the instruments, 62 analyses of differences between format mean scores were made, and significant differences were found in 10 (16%) of the 62. Limiting the analysis to the 17 studies with adequate power to analyze mean differences, 6 differences were found in 31 (19%) analyses. Including studies of all sample sizes, significant mean differences between scores were found in 8 of the 40 investigated instruments. The effects sizes (Cohen’s *d*) of the mean score differences ranged from .14 to .98, showing that some of the effects were large.

To assess whether there was an aggregate trend in the data, both studies reporting significant differences and those reporting nonsignificant but consistent numerical differences were analyzed (ie, including analyses that showed higher but non-significant values for either condition). In total, 30 instruments or subscales reported a higher mean score for the PnP version, and 26 reported a higher mean score for the digital version. This difference in proportion was not significant (*P*=.26).

Correlations between format scores were reported for 28 instruments and ranged between *r=*.35 and *r=*.99. More than half of the instruments (16/33, 57%) showed strong uncontradicted correlations (>.80) between format scores, while the correlations between formats scores were ambiguous for five instruments. Strong interformat correlations have been replicated for only four instruments: Agoraphobic Cognitions Questionnaire (ACQ), Mobile Inventory (MI), Beck Depression Inventory II (BDI-II), and Montgomery-Asberg Depression Rating Scale-Self-report (MADRS-S). Two studies investigated differences in factor structure and model fit between PnP-format and digital format of questionnaires, and neither of these studies found any significant model differences. See Multimedia Appendix 4 for a review of the extracted psychometric properties.

The reported significant mean differences between format scores were not evenly distributed among the studies. Instead, many of the reported differences could be found in a small number of studies. All the 10 significant mean differences found were reported in five studies, and one particular study reported 5 (50%) of the identified differences. The five studies did not differ markedly from the other studies concerning study characteristic (eg, publication year, sample size, and quality assessment score). The sample size in the five studies that reported inequality between administration formats ranged from 83 to 1171, with two studies having enough power to detect a small effect size. In conclusion, none of the study characteristics assessed in this study could explain the reported inequalities between formats found in these studies.

Differences in mean scores from the respective format were found in ACQ, Beck Anxiety Inventory (BAI), BDI-II (twice), Body Sensations Questionnaire (BSQ) (twice), Center for Epidemiologic Studies Depression Scale (CES-D), MI, SCL-90-R, and State-Trait Anxiety Inventory-State (STAI-S). Of these instruments, ACQ, BAI, BDI-II, CES-D, and SCL-90-R were also investigated in other studies with sufficient power to detect effects of corresponding sizes without finding significant mean differences, making results regarding interformat reliability contradictory (see Multimedia Appendix 4). BSQ and MI have either not been repeatedly investigated or been investigated in studies with insufficient power.

### Test-Retest

Test-retest analysis for digital formats was conducted for 14 instruments in six studies, and the mean correlation between test occasions was *r*=.84 (SD .07, range *r*=.70-.90). The majority of the instruments (10/14, 71%) showed good test-retest reliability (>.80). Further, no significant effects of time were reported for the seven instruments analyzed with two-way ANOVA.

### Internal Consistency

Internal consistency for the digital version of the instruments was reported in 24 studies and for 26 different instruments. Cronbach alpha was calculated for the digital format of an instrument in 69 instances and the mean value was .87 (SD .09, range .52-.97). A large majority of the instruments (24/26, 94%) showed adequate internal consistency (alpha>.70). Questionable internal consistencies were found for 12-Item Short Form Health Survey (SF-12 v2), Mental Component Scale, and for subscales of SCL-90-R, and the alpha results regarding Insomnia Severity Index were ambiguous.

## Discussion

### Principal Findings

This review aimed to investigate the interformat reliability of self-report instruments used in psychotherapy research. Studies comparing digital and PnP formats of 40 different instruments, covering various psychiatric disorders, were identified in the review process. Similar to a previous study in the somatic field, this systematic review of the literature showed that generally the reliability between digital and pen and paper versions of instruments is high [12]. The large majority of studies found adequate correlations between format scores and no significant differences between means derived from the respective format. For example, high quality studies consistently report a high interformat reliability for MADRS-S showing that this instrument can be used with confidence in online psychotherapy research. Several other well-known instruments, such as the Alcohol Use Disorder Identification Test (AUDIT), PCL-C, and Patient Health Questionnaire (PHQ-9) have also shown high interformat reliability, but the results have not yet been replicated in high quality studies. While most instruments have been investigated only once, some instruments, notably the BDI and the CES-D, have been investigated multiple times. Generally, these studies support the interformat reliability of the BDI and the CES-D. In contrast, the instruments SCL-90-R and GHQ-28 showed less than satisfactory reliability in several studies. The reasons for this are unknown, but both instruments are rather complex and contain several subscales that are designed to capture many different domains of psychological health. It is possible that either the complexity or the broader psychiatric scope of these instruments, compared to most others in this review, make them sensitive to a change of format.

Significant differences in mean scores between formats were found in 16% of all analyses. These differences were found in a small number of studies, indicating that the results may be due to study or participant characteristics rather than the properties of the instruments. The effects sizes of the mean differences ranged from small to large, indicating that the significant differences were not just a matter of high power in studies with large samples. It is noteworthy that several high powered studies did not find significant differences between format scores. At the same time, as exemplified by the largest study included in this review, by Yu & Yu [47], the effect may often be too small to be detected in smaller studies. Such small effects of instrument format would not have any major implications for most psychotherapy research but may, for example, affect the results of prevalence studies. Also, some of the differences found between format means were of medium or large effect sizes, which implies that study design may affect the results substantially. If researchers are not careful and meticulous in designing their studies, the results from computer-based psychotherapy may potentially not be comparable to that of traditional psychotherapy.

The range of correlations between formats was wide, from .35 to .99. The lowest correlations were found in certain subscales of the general health questionnaires, scales that overall show lower reliability, and the STAI-S, an instrument that explicitly measures the current condition and may thus be very sensitive to time effects. Interestingly, significant format differences were reported for the BDI, MI, and ACQ, while these instruments at the same time reported high correlations. This underscores the importance of not solely relying on correlations when assessing interformat reliability.

When performing a systematic review, there is always the risk of selection bias when searching for and including relevant studies. This includes publication bias, which automatically narrows the range of studies that can be included but is also relevant when perusing reference lists of identified papers for additional studies. However, these risks may be somewhat lower in the case of studies on interformat reliability since both positive and negative results should draw attention from publishers. Still, the risk of bias in the included studies in any review should not be underestimated.

The majority of studies were of high scientific quality, using adequate designs and statistical analyses. However, only half of the studies had an adequate sample size to detect mean differences with a medium effect size and power calculations were seldom reported. Also, the time interval between data collections, arguably a very important factor, was not reported in all studies using a crossover design. Possible interval differences may thus explain some of the variance found between studies. Future studies should focus on improving the methodological quality by increasing the sample size to achieve sufficient power. Another recommendation is that studies report both interformat correlation and differences between format means.

Knowledge about factors that may affect interformat reliability is still limited. One potential factor is the characteristics of the digital format itself. The layout, user interface, etc, are likely to affect the score of an instrument in some way, at least if these characteristics are markedly different from the PnP version. It is valuable to know the degree of similarity between the PnP and the digital version in order to assess this potential effect. Regrettably, few studies report what adaptations are made when transferring the instrument to the new administration format.

In this review, only three studies used digital formats other than computer/Internet: Cooke et al [22] used a palm device, Swartz et al [39] used a personal digital assistant, and Bush et al [17] used online and smartphone formats. None of these studies reported any significant differences of mean values between formats, at least indicating that interformat reliability could be stable over different forms of digital platforms.

Further, respondents’ reactions to the digital format may affect results if the digital medium is perceived as different, for example, as more secure or anonymous than the traditional pen and paper medium. In research on survey methodology, a number of studies have shown that data collected on the Internet can be equivalent to data collected with traditional methods [53-55]. While there are no empirical studies investigating how participants react to instruments in psychotherapy research, the conclusions in the present review are in line with these results.

### Limitations

This study has some limitations. Only instruments typically used in psychotherapy research were included. Psychiatric symptom scales of the questionnaire type may be less sensitive to administration differences than some other measurements, such as VAS-scales or behavioral diaries. It is thus unclear whether the results of this study could be generalized to these other types of measurements as well. Further, no effort was made to contact authors for additional data if relevant information was missing in the articles included. Since several studies failed to report variables that were investigated in this study, contact with authors may have contributed more data. Finally, since only reference lists from included studies were perused, it is possible that we missed studies that were cited in papers that we reviewed but excluded. A strength of the current study was the effort to find and include older studies as well as studies with different designs.

While this review focused on reliability of digital instruments, future studies could also investigate aspects of validity. It could be argued that instruments showing adequate interformat reliability do not need to confirm the validity for digital use if validity is already established for their pen and paper versions. This is, however, an empirical question and could be important to consider. To our knowledge, few studies have investigated the validity of digital instruments.

In general, instruments used in Internet-based psychotherapy research show high interformat reliability and can be used with confidence. There are also some signs that the factor structure is not affected by delivery format. There is, however, still a need for well-designed and high powered studies investigating the most widely used instruments, such as the PHQ-9. While the use of mobile technology will increase, very few studies have investigated instruments administrated through a smartphone, tablet computer, or similar device. Future studies could thus focus on these platforms.

However, even within platforms, such as personal computer or smartphone, there is almost limitless variation when it comes to instrument presentation. The format per se may be much less important than the specific presentation. Different presentations and adaptations of instruments could, in the future, be investigated experimentally to identify factors that influence interformat reliability. Since few studies report in any detail what adaptations they have made of the digital instruments, effect of presentation is still largely unknown. One of the benefits of digital instruments is the possibility to design smart questionnaires that adapt to the respondent’s answers. While outside the scope of the present review, such development may be more relevant for clinical care in the future [56].

### Conclusion

This review concludes that, while instruments in most studies show high interformat reliability, there are some exceptions, and it is still unclear if these exceptions are due to psychometric properties of specific instrument or to study properties. In general, instruments used in psychotherapy research seem to be robust over administration formats. Future studies should increase sample sizes and both investigate and clearly report how digital adaptation of instruments are made.

## Multimedia Appendix 1

Study quality assessment.

## Multimedia Appendix 2

PRISMA flow chart.

## Multimedia Appendix 3

Study and participant characteristics.

## Multimedia Appendix 4

Psychometric results from all studies.

## Abbreviations

ACQ: Agoraphobic Cognitions Questionnaire
ANOVA: analysis of variance
AUDIT: Alcohol Use Disorder Identification Test
BAI: Beck Anxiety Inventory
BDI: Beck Depression Inventory
BSQ: Body Sensations Questionnaire
CES-D: Center for Epidemiologic Studies Depression scale
GHQ: General Health Questionnaire
MADRS-S: Montgomery–Asberg Depression Rating Scale Self-report
MI: Mobility Inventory
PCL-C: PTSD Check List–Civilian Version
PHQ: Patient Health Questionnaire
PnP: pen and paper
PRISMA: Preferred Reporting Items for Systematic Reviews and Meta-Analyses
SCL-90-R: Symptom Checklist 90 Revised
SF12V2: Short Form (12) Health Survey Version Two
STAI-S: State-Trait Anxiety Inventory–State
STAI-T: State-Trait Anxiety Inventory–Trait
VAS: Visual Analogue Scale
