# Supplementary material for: Interformat Reliability of Digital Psychiatric Self-Report Questionnaires: A Systematic Review
Source: J Med Internet Res. 2014 Dec 3;16(12):e268. doi: 10.2196/jmir.3395 (PMC4275488; doi:10.2196/jmir.3395)
Supplement: Supplementary file 1 [file jmir_v16i12e268_app1.pdf]

Study quality assessment of one sample design studies

Study	Analysis	Randomized	Report of statistics	Sample size	Sample type	Adaptation	Sum
Austin et al (2006)	2	2	2	1	2	0	9
Brock et al (2012)	2	0	2	2	1	0	7
Bush et al (2013)	1	2	2	2	2	0	9
Butler et al (1988)	1	2	1	2	0	1	7
Carlbring et al (2007)	2	2	2	2	2	0	10
Chan-Pensley (1999)	2	0	1	1	2	0	6
Coles et al (2007)	2	2	2	1	1	1	9
Cook et al (2007)	1	2	2	2	2	2	11
Fortson et al (2006)	2	1	2	2	1	0	8
George et al (1992)	2	2	2	1	1	1	9
Glaze & Cox (1991)	1	1	1	2	2	1	8
Herrero & Meneses (2006)	2	2	2	2	1	0	9
Hirai et al (2011)	2	2	2	2	0	0	8
Holländare et al (2008)	2	2	2	1	0	0	7
Holländare et al (2010)	2	2	2	1	2	1	10
Kurt et al 2004	1	0	2	2	2	2	9

Lankford et al (1994)	2	2	2	2	1	0	9
Lukin et al (1985)	2	2	2	1	1	1	9
Miller et al (2002)	2	2	2	2	1	0	9
Murelle et al (1992)	1	0	1	2	1	1	6
Ogles et al (1998)	1	0	2	2	1	2	8
Read et al (2008)	1	0	2	2	1	0	6
Schmitz et al (2000)	2	2	2	2	2	2	12
Schulenberg & Yutrzenka (2001)	2	2	2	2	1	0	9
Swartz et al (2007)	2	2	2	2	2	1	11
Thorén et al (2012)	2	2	2	1	2	0	9
Thorndike et al (2011)	2	2	2	1	2	0	9
Vallejo et al (2008)	2	2	2	1	1	2	10
Vallejo et al (2007)	2	0	2	1	1	0	6
Whitehead (2011)	2	2	2	2	1	1	10
Wijndaele et al (2007)	1	0	2	2	2	2	9
Zimmerman & Martinez (2012)	2	0	2	1	2	1	8
Yu & Yu	1	2	2	2	1	0	8

Study assessment criteria for scoring.

**Aspect**	**Scoring**
Analysis<br>What type of analysis was used to asses inter format reliability?	2 = Comparison of mean difference (e.g., t-test, ANOVA), 1= Correlation or model test only, 0 = Unclear.
Randomized<br>Were participants randomized to conditions (depending on design)?	2 = Yes, 0 = No.
Report of statistics<br>Are relevant statistics reported?	2 = Mean and SD for scales, t-values and p-values for sig dif are reported, 1 = Is lacking any of these, 0 = Lack several of these.
Sample size<br>Was the sample size adequate for the statistical analyses?	2 = Adequate sample size or above (n>20 for correlation (α=.05, β=.20, r=.6) analysis, n>128 for t-tests and ANOVA (α=.05, β=.20 and medium effect size) analysis), 1 = 51-99% of recommended, 0 = 0-50% of recommended.
Sample type<br>Was the study group used for adequate psychometric evaluation of the particular instrument?	2 = Clinical instrument used in adequate clinical group or a screening instrument used in a clinical or non restricted sample, 1 = clinical instrument used in non-clinical sample or screening instrument used in restricted sample (e.g., student sample), 0 = clinical instrument used in non clinical restricted sample.
Adaptation<br>Were the layout, text format, response options, presentation of items, and possibilities to interact in the digital format of the instrument described?	2 = >3 features described, 1 = 2-3 features described, 0 = 0-1 features described.

Note. If inadequate or no information is available for a certain aspect a score of 0 is given.
