# Supplementary figures and images for: Interformat Reliability of Digital Psychiatric Self-Report Questionnaires: A Systematic Review
Source: J Med Internet Res. 2014 Dec 3;16(12):e268. doi: 10.2196/jmir.3395 (PMC4275488; doi:10.2196/jmir.3395)

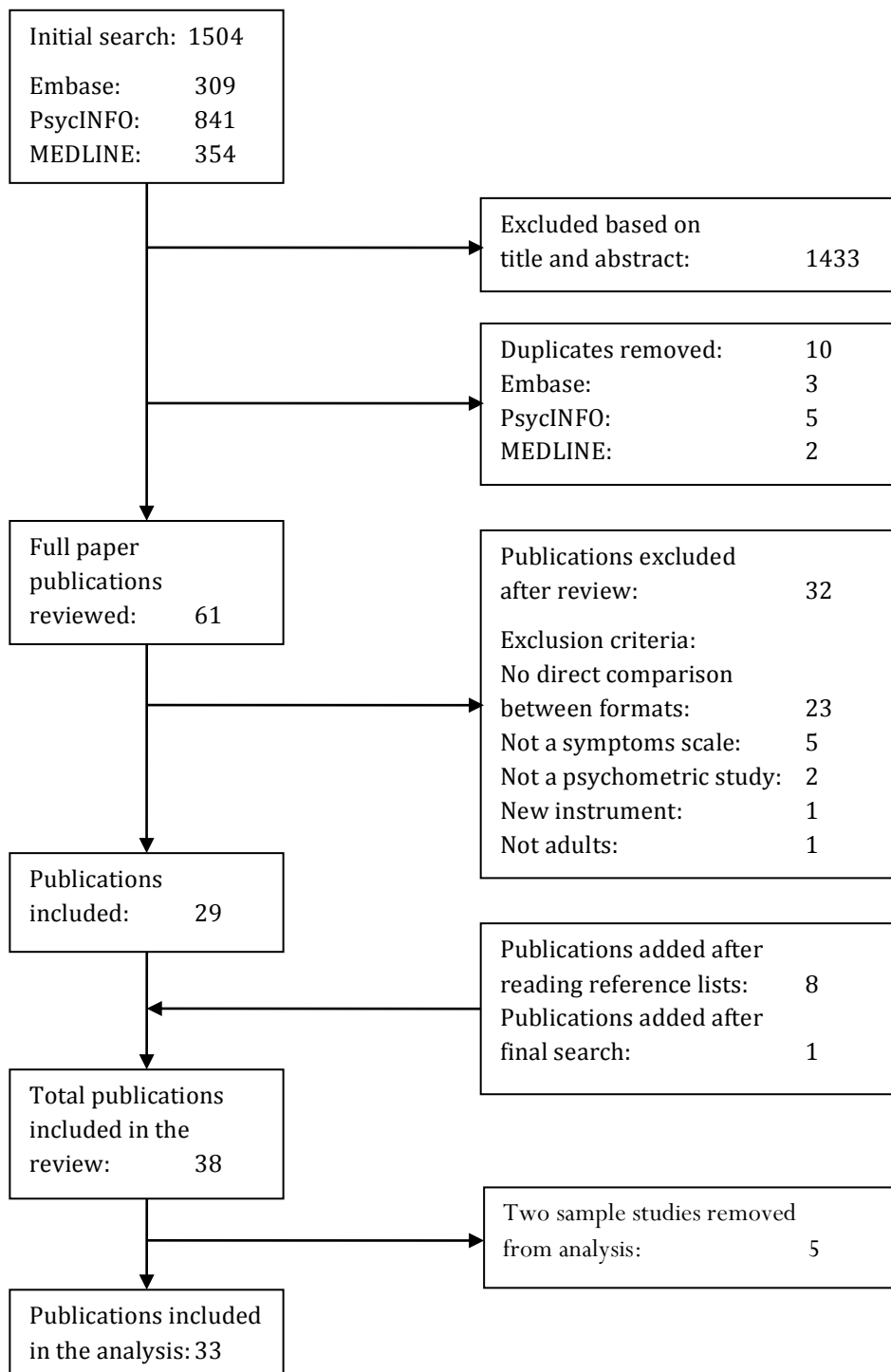

Supplement: Supplementary file 2 [file jmir_v16i12e268_app2.pdf]
