# Supplementary material for: Interformat Reliability of Digital Psychiatric Self-Report Questionnaires: A Systematic Review
Source: J Med Internet Res. 2014 Dec 3;16(12):e268. doi: 10.2196/jmir.3395 (PMC4275488; doi:10.2196/jmir.3395)
Supplement: Supplementary file 3 [file jmir_v16i12e268_app3.pdf]

Study and participant characteristics of one sample design studies

**Publication**	**Instruments**	**Formats**[a]	**Design**	**Sample size**	**Sample type**	**Age mean (SD)**	**Gender (% women)**	**Computer experience**
Austin et al (2006)	BSQ, ACQ, MI	PnP and Online	Cross over (2x2)	110	Panic disorder	38.50 (11.3)	70%	N/a
Brock et al (2012)	CES-D, BAI	PnP and Online	Cross over (4x2)	169	Students	18.84 (1.6)	79.9%	N/a
Bush et al (2013)	PCL-C, PHQ-9	PnP, online and smartphone	Randomized	45	Soldiers	Not reported	23,9%	Assessed
Butler et al (1988)	SCANS	PnP and Computer	Cross over (2x2)	47	N/a	24.62 (5.4-4.8)	66.0%	N/a
Carlbring et al (2007)	BSQ, ACQ, MI, BAI, BDI-II, MADRS-S	PnP and Online	Cross over (2x2)	344	Panic disorder	37.6 (10.9)	49%	Assessed
Chan-Pensley (1999)	AUDIT	PnP and Computer	Cross over (2x2)	110	Alcohol abuse /dependence	43.06 (10.6)	39.1%	N/a
Coles et al (2007)	OCI, OBQ-44	PnP and Online	Cross over (2x2)	105	Students	18.93 (1.1), 19.08 (1.7)	76.9%	Assessed

Cook et al (2007)	QIDS-SR	PnP and Palm	Cross over (2x2)	80	Depression	44.1 (11.5)	69%	Assessed
Fortson et al (2006)	CES-D, TSS	PnP and Online	Cross over (2x2)	411	Students	20.0 (3.2)	56%	Assessed
George et al (1992)	BDI, STAI-S, STAI-T	PnP and Computer	Randomized	97	Students	Median=19	53.6%	N/a
Glaze & Cox (1991)	EPDS	PnP and Computer	Cross over (2x2)	29	Postpartum women	N/a	100%	N/a
Herrero & Meneses (2006)	CESD-7	PnP and Online	Randomized	530	Students	29.06 (5.1)	66%	N/a
Hirai et al (2011)	SIAS, SPS	PnP and Online	Randomized	514	Students	20.2 (3.3)	64%	N/a
Holländare et al (2008)	BDI II, MADRS-S	PnP and Online	Cross over (2x2)	71	Students	N/a	31.8%	N/a
Holländare et al (2010)	BDI II, MADRS-S	PnP and Online	Cross over (2x2)	87	Depression	41.1 (13.0)	65.5%	N/a
Kurt et al (2004)	CESD-R 20, GDS 15	PnP and Computer	Cross over (2x2)	53	Elderly patients in primary care	N/a	64.8%	Self-rated
Lankford et al (1994)	BDI, STAI	PnP and Computer	Randomized	131	Students	N/a	49%	N/a
Lukin et al	STAI, BDI	PnP and	Cross over	66	Students	N/a	66.7%	N/a

(1985)		Computer	(2x2)					
Miller et al (2002)	AUDIT, ADS, RAPI	PnP and Online	Randomized	255	Students	20.9 (2.0)	64%	N/a
Murelle et al (1992)	MAST, CAGE, FTQ, CES-D, EAT, DAST, STAI	PnP and Computer	Non randomized	83	Students	20.6 (1.8)	70%	N/a
Ogles et al (1998)	CES-D	PnP and Computer	Non randomized	113	Mixed community sample	41.6 (14.1)	71%	N/a
Read et al (2008)	PCL-C, TLEQ	PnP and Online	Non randomized	38	Students with traumatic experience and one PTSD symptom	19.6 (1.5)	50%	N/a
Schmitz et al (2000)	SCL-90-R	PnP and Computer	Randomized	282	Psychosomatic outpatients	38.7 (11.7) 38.9 (12.2)	62.1%	N/a
Schulenberg & Yutrzenka (2001)	BDI-II	PnP and Computer	Cross over (4x2)	180	Students	21.95 (5.5)	78.3%	Assessed
Swartz et al (2007)	CES-D	PnP and PDA	Cross over (2x2)	756	Cancer patients	55 (13)	53%	N/a
Thorén et al (2012)	HADS	PnP and Online	Cross over (2x2)	53	Hearing impaired	68.3 (11.3)	24.5%	N/a

					adults			
Thorndike et al (2011)	ISI	PnP and Online	Cross over (2x2)	43	Insomnia	45.02 (11.1)	76.8%	N/a
Vallejo et al (2007)	GHQ-28, SCL-90-R	PnP and Online	Non randomized	100	Students	27.4 (10.0)	78%	N/a
Vallejo et al (2008)	GHQ-28, SCL-90-R	PnP and Online	Cross over (2x2)	40	Students	22.2 (N/a)	60%	Assessed
Whitehead (2011)	HADS, SF-12	PnP and Online	Randomized	970	Students	24.07 (8.5)	N/a	N/a
Wijndaele et al (2007)	GHQ-12, SCL-90-R	PnP and Online	Non randomized	245	Community sample	N/a	50.8%	Assessed
Yu & Yu (2007)	CES-D	PnP and Online	Randomized	1171	Teachers	N/a	N/a	N/a
Zimmerman & Martinez (2012)	CUDOS	PnP and Online	Non randomized	53	Depression	45.1 (12.3)	75.5%	N/a

a. PnP, computer, online or palm/cell phone

Note. N/a = Not assessed or Not available
