# Supplementary material for: Interformat Reliability of Digital Psychiatric Self-Report Questionnaires: A Systematic Review
Source: J Med Internet Res. 2014 Dec 3;16(12):e268. doi: 10.2196/jmir.3395 (PMC4275488; doi:10.2196/jmir.3395)
Supplement: Supplementary file 4 [file jmir_v16i12e268_app4.pdf]

Psychometric results from all studies.

**Study**	**Instrument**	**Format correlation**[a]	**Means (SD)**		**Format difference**	**Test-retest Reliability**[b]	**Internal consistency**[b] (Cronbach's alpha)
			PnP	Digital			
Austin et al (2006)	BSQ	ICC=.73	47.96 (13.03)	49.22 (12.37)	F=6.97, p<.05, *d*=.51[d]	N/a	.86
			55.67 (10.02)	48.34 (12.07)			
			50.69 (15.50)	52.08 (9.02)			
			52.84 (9.35)	49.00 (10.91)			
	ACQ	ICC=.92	30.74 (8.50)	31.11 (8.20)	N.s.	N/a	.90
			33.69 (11.17)	33.44 (11.46)			
			50.58 (7.14)	48.96 (6.36)			
			47.80 (7.95)	47.37 (8.62)			
	MI Accompanied	ICC=.95	49.92 (25.26)	50.74 (26.20)	N.s.	N/a	.95
			55.55 (23.43)	53.90 (21.17)			
			59.06 (19.43)	59.83 (17.85)			
			63.50 (20.44)	63.30 (21.67)			
	MI Alone	ICC=.96	60.59 (29.43)	60.59 (28.61)	N.s.	N/a	.96
			69.69 (32.29)	70.14 (30.49)			
			75.36 (25.03)	75.21 (23.92)			
			80.72 (22.34)	83.37 (24.24)			

Brock et al (2012)	CES-D	N/a	12.00 (7.09)	12.17 (7.75)	N.s.	ICC=.84	N/a
			12.58 (9.04)	14.85 (8.85)			
	BAI	N/a	8.55 (6.87)	6.21 (6.42)	N.s.	ICC=.84	N/a
			9.08 (8.72)	9.43 (6.96)			
Bush et al (2013)	PCL-C	Cell phone: ICC= .90<br>Online: ICC=.95	29.6 (13.4)	Cell phone: 32.2 (14.5)<br>Online: 29.3 (13.4)	N.s.[c]	Cell phone: ICC= .91	Cell phone: .95<br>Online: .96
	PHQ-9	Cell phone: ICC= .92<br>Online: ICC=.92	5.9 (5.6)	Cell phone: 5.2 (5.2)<br>Online: 5.1 (4.9)	N.s.[c]	Cell phone: ICC= .93	Cell phone: .87<br>Online: .85
Butler et al (1988)	SCANS	N/a	N/a	N/a	N/a	N/a	N/a
	SCANS subscales	r=.77-.97	N/a	N/a	N/a	N/a	N/a
Carlbring et al (2007)	BSQ	r=.81	3.02	2.90	F=36.5, p<.001, *d*=.65[d]	N/a	.84/.87
	ACQ	r=.89	2.53	2.42	F=45.4, p<.001, *d*=.73[d]	N/a	.81/.83
	MI Accompanied	r=.96	2.06 (0.81)	2.10 (0.77)	N.s.	N/a	.95/.94
			2.07 (0.77)	2.04 (0.77)			
	MI Alone	r=.95	2.68	2.71	F=5.9, p<.05, *d*=.26[d]		.94/.94
	BAI	r=.84	22.62	19.63	F=82.2, p<.01,	N/a	.88/.91

					d=.98[d]		
	BDI-II	r=.94	17.52	18.01	F=6.3, p<.05, *d*=.27[d]	N/a	.88/.89
	MADRS-S	r=.91	16.69 (7.4)	16.42 (7.1)	N.s.	N/a	.82/.83
			17.11 (9.4)	16.79 (8.3)			
Chan-Pensley (1999)	AUDIT	N/a	29.72 (6.51)	29.95 (6.72)	N.s.	N/a	N/a
Coles et al (2007)	OCI	r=.89	16.75 (18.16)	15.76 (17.26)	N.s.	N/a	.94
	OBQ-44	r=.83	121.49 (46.43)	123.51 (48.53)	N.s.	N/a	.97
Cook et al (2007)	QIDS-SR	ICC=.99	15.1 (5.2)	15.3 (5.2)	N.s.[c]	N/a	N/a
Fortson et al (2006)	CES-D	N/a	13.81 (8.89)[e]	12.34 (8.59)[e]	N.s.	N/a	.88/.89
	TSS Frequency	N/a	65.03 (20.54)[e]	67.20 (21.35)[e]	N.s	N/a	.94/.95
	TSS Distress	N/a	63.97 (22.62)[e]	64.36 (23.29)[e]	N.s		.96
George et al (1992)	BDI	N/a	6.02 (5.17)	8.21 (4.69)	t=2.18, p<.05, *d*=.44[d]	N/a	N/a
	STAI-S	N/a	34.88 (7.03)	38.69 (9.61)	t=2.23, p<.05, *d*=.45[d]	N/a	N/a
	STAI-T	N/a	40.20 (6.28)	40.77 (8.35)	N.s.	N/a	N/a
Glaze & Cox (1991)	EPDS	r=.98	13.34 (7.60)[e]	13.59 (7.75)[e]	N.s.[c]	N/a	N/a

Herrero & Meneses (2006)	CESD-7	N/a	11.85 (3.78)	11.57 (3.79)	N.s.	N/a	.82
Hirai et al (2011)	SIAS	N/a	20.5 (12.39)	20.0 (13.23)	N.s.	N/a	.93
	SPS	N/a	15.6 (10.68)	16.4 (12.66)	N.s.	N/a	.93
Holländare et al (2008)	BDI-II	r=.94	8.41 (9.22)[e]	8.18 (9.84)[e]	N.s.	N/a	.95
	MADRS-S	r=.92	7.23 (7.53)[e]	7.36 (7.50)[e]	N.s.	N/a	.90
Holländare et al (2010)	BDI II	r=.89	30.55 (10.72)	29.68 (10.07)	N.s.	N/a	.87/.89
	MADRS-S	r=.84	24.43 (6.97)	23.79 (7.98)	N.s.	N/a	.73/.81
Kurt et al (2004)	GDS-15	r=.72/.83	17.68 (2.48)[e]	17.59 (2.38)[e]	N.s.[c]	r=.70	N/a
	CESD-R 20	r=.61/.74	10.19 (14.11)[e]	10.59 (10.85)[e]	N.s.[c]	r=.85	N/a
Lankford et al (1994)	BDI	N/a	5.72 (3.83)	6.32 (4.34)	N.s. effect of format.	N/a	N/a
Lukin et al (1985)	STAI-T	N/a	46.35 (6.77)	46.06 (8.23)	N.s.	N.s. effect of time.	N/a
	BDI	N/a	7.68 (5.88)	7.67 (5.84)	N.s.	N.s. effect of time.	N/a
Miller et al (2002)	ADS	N/a	6.95 (4.56)[e]	5.90 (3.72)[e]	N.s.	r=.84	N/a
	AUDIT	N/a	4.45 (3.35)[e]	4.40 (3.41)[e]	All n.s.	r=.92	N/a

	RAPI 1 month	N/a	1.45 (2.85)[e]	1.50 (2.75)[e]	N.s.	r=.78	N/a
	RAPI 6 months	N/a	4.15 (5.25)[e]	3.85 (5.77)[e]	N.s.	r=.89	N/a
	RAPI 1 year	N/a	7.35 (8.01)[e]	5.85 (7.83)[e]	N.s.	r=.88	N/a
Murelle et al (1992)	MAST	r=.73	N/a	N/a	N/a	N/a	N/a
	CAGE	r=.89	N/a	N/a	N/a	N/a	N/a
	DAST	r=.65	N/a	N/a	N/a	N/a	N/a
	CES-D	r=.54	N/a	N/a	N/a	N/a	N/a
	STAI	r=.35	N/a	N/a	N/a	N/a	N/a
	EAT	r=.63	N/a	N/a	N/a	N/a	N/a
	FTQ	r=.68	N/a	N/a	N/a	N/a	N/a
Ogles et al (1998)	CES-D	r=.96	N/a	N/a	N/a	N/a	.91
Read et al (2008)	PCL-C	r=.68	34.21 (12.06)	30.56 (12.44)	N.s.	N/a	.91
	TLEQ[f]	r=.85	3.81 (2.95)	3.95 (2.82)	N.s.	N/a	N/a
Schmitz et al (2000)	SCL-90-R	N/a	1.20 (0.66)	1.29 (0.66)	N.s.	N/a	.97
	SCL-90-R subscales				Sig. diff between formats on two subscales.		.74-.90
Schulenberg & Yutrzenka (2001)	BDI-II	r=.98	8.83 (6.80)	10.09 (9.08)	N.s.	N/a	.91

			8.91 (8.41)	9.08 (7.39)			
Swartz et al (2007)	CES-D	N/a	15.1	13.6	N.s. effect of format. Sig. format x order interaction effect.	N/a	
Thorén et al (2012)	HADS	r=.67	7.3 (5.9)	6.6 (5.4)	N.s.	N/a	.85
Thorndike et al (2011)	ISI	r=.98/.99	15.86 (3.80)	16.00 (3.87)	N.s.	N/a	.61/.88
			11.03 (6.40)	10.91 (6.20)			
Vallejo et al (2008)	GHQ-28	r=.84	16.13[e] (13.80)	20.45[e] (13.47)	N.s.[c]	N/a	.91
	GHQ-28 subscales	r=.49-.92			Sig. effect of format on one subscale. Sig. format x order interaction effect on one subscale.		
	SCL-90-R	r=.88	.73 (.62)[e]	.65 (.58)[e]	N.s.	N/a	.97
	SCL-90-R subscales	r=.74-.96			Sig. effect of format on two subscales. Sig. time x format interaction effect on three		

					subscales.		
Vallejo et al (2007)	SCL-90-R	r=.83	.50 (.40)	.37 (.36)	t= 5.47, p< .001, *d*=.34[d]	N/a	.97
	SCL-90-R subscales	r=.63-.86			Sig. diff. between formats on 7 subscales.		
	GHQ-28	r=.69	17.71 (9.13)	16.63 (9.00)	N.s.	N/a	.90
	GHQ-28 subscales	r=.30-.72			Sig. diff. between formats on one subscale.		
Whitehead (2011)	HADS-Anxiety	N/a	6.31 (3.72)	6.39 (3.68)	N.s.	N/a	.80
	HADS-Depression	N/a	3.24 (3.05)	3.52 (3.04)	N.s.	N/a	.76
	SF12v2 MCS	N/a	46.28 (10.02)	46.04 (9.67)	N.s.	N/a	.66
	SF12v2 PCS	N/a	54.62 (6.7)	54.13 (7.2)	N.s.	N/a	.93
Wijndaele et al (2007)	GHQ-12	ICC=.75	N/a	N/a	N/a	ICC=.76	.79
	SCL-90-R	N/a	N/a	N/a	N/a	N/a	N/a
	SCL-90-R subscales	ICC=.54-.81				ICC=.58-.85	.52-.94
Zimmerman & Martinez (2012)	CUDOS	ICC=.96	20.0 (14.6)	20.6 (13.9)	N.s.	N/a	.93
Yu & Yu	CES-D	N/a	12.14 (8.02)	11.03 (7.87)	t=2.39, p=.02[c],	N/a	N/a

(2007)					*d*=.14[d]<br>Invariant factor structure between formats.		

Note. Results are shown for total sample in studies with many groups. a = ICC = Intra Class Correlation and r = Pearson's r, b = digital version, c = t-tests conducted and interpreted by the authors of the present study based on values from original article, d = effect sizes calculated by the authors of the present study based on values from the original article, e = mean score calculated and standard deviation estimated by the authors of the present study based on values from the original article, f = shortened web-version.
